# Prognostic accuracy of SOFA, qSOFA and SIRS criteria in hematological cancer patients: a retrospective multicenter study

**DOI:** 10.1186/s40560-019-0396-y

**Published:** 2019-08-07

**Authors:** Lucie Probst, Enrico Schalk, Tobias Liebregts, Vanja Zeremski, Asterios Tzalavras, Michael von Bergwelt-Baildon, Nina Hesse, Johanna Prinz, Jörg Janne Vehreschild, Alexander Shimabukuro-Vornhagen, Dennis A. Eichenauer, Jorge Garcia Borrega, Matthias Kochanek, Boris Böll, for the Working Party on Intensive Care Medicine in Hematologic and Oncologic Patients (iCHOP) of the German Society of Hematology and Medical Oncology (DGHO)

**Affiliations:** 1 University of Cologne, Department I of Internal Medicine, Center for Integrated Oncology Aachen Bonn Cologne Duesseldorf, Cologne, Germany; 2 Department of Hematology and Oncology, Otto-von-Guericke University, Magdeburg, Germany; 3 Department of Bone Marrow Transplantation, West German Cancer Center, University Hospital Essen, University of Duisburg-Essen, Essen, Germany; 4 Department of Medicine III, University Hospital LMU Munich, Munich, Germany; 5 German Centre for Infection Research, partner-site Bonn-Cologne, Cologne, Germany; 6 Medical Department 2, Hematology/Oncology, Goethe University of Frankfurt, Frankfurt, Germany

**Keywords:** Sepsis, SIRS, Sepsis-3, qSOFA, Cancer, Hematological malignancies

## Abstract

**Background:**

With Sepsis-3, the increase in sequential organ failure assessment (SOFA) as a clinical score for the identification of patients with sepsis and quickSOFA (qSOFA) for the identification of patients at risk of sepsis outside the intensive care unit (ICU) were introduced in 2016. However, their validity has been questioned, and their applicability in different settings and subgroups, such as hematological cancer patients, remains unclear. We therefore assessed the validity of SOFA, qSOFA, and the systemic inflammatory response syndrome (SIRS) criteria regarding the diagnosis of sepsis and the prediction of in-hospital mortality in a multicenter cohort of hematological cancer patients treated on ICU and non-ICU settings.

**Methods:**

We retrospectively calculated SIRS, SOFA, and qSOFA scores in our cohort and applied the definition of sepsis as “life-threatening organ dysfunction caused by dysregulated host response to infection” as reference. Discriminatory capacity was assessed using the area under the receiver operating characteristic curve (AUROC).

**Results:**

Among 450 patients with hematological cancer (median age 58 years, 274 males [61%]), 180 (40%) had sepsis of which 101 (56%) were treated on ICU. For the diagnosis of sepsis, sensitivity was 86%, 64%, and 42% for SIRS, SOFA, and qSOFA, respectively. However, the AUROCs of SOFA and qSOFA indicated better discrimination for sepsis than SIRS (SOFA, 0.69 [95% CI, 0.64–0.73] *p* < 0.001; qSOFA, 0.67 [95% CI, 0.62–0.71] *p* < 0.001; SIRS, 0.57 [95% CI, 0.53–0.61] *p* < 0.001).

In-hospital mortality was 40% and 14% in patients with and without sepsis, respectively (*p* < 0.001). Regarding patients with sepsis, mortality was similar in patients with positive and negative SIRS scores (39% vs. 40% (*p* = 0.899), respectively). For patients with qSOFA ≥ 2, mortality was 49% compared to 33% for those with qSOFA < 2 (*p* = 0.056), and for SOFA 56% vs. 11% (*p* < 0.001), respectively. SOFA allowed significantly better discrimination for in-hospital mortality (AUROC 0.74 [95% CI, 0.69–0.79] *p* < 0.001) than qSOFA (AUROC 0.65 [95% CI, 0.60–0.71] *p* < 0.001) or SIRS (AUROC 0.49 [95% CI, 0.44–0.54] *p* < 0.001).

**Conclusions:**

An increase in SOFA score of ≥ 2 had better prognostic accuracy for both diagnosis of sepsis and in-hospital mortality in this setting, and especially on ICU, we observed limited validity of SIRS criteria and qSOFA in identifying hematological patients with sepsis and at high risk of death.

**Electronic supplementary material:**

The online version of this article (10.1186/s40560-019-0396-y) contains supplementary material, which is available to authorized users.

## Background

Sepsis is among the leading causes of death with an estimated annual global incidence of 31.5 million cases of sepsis and 19.4 million cases of severe sepsis [1]. Although outcomes have improved due to collaborative efforts [2], mortality remains high with estimated in-hospital mortality rates of 17–26%. In patients presenting with septic shock, the estimated mortality is even higher with rates of 46% and higher depending on the definition [3].

Accurate definition, diagnosis, and early recognition of sepsis are crucial for the effective management of patients, as it improves outcomes [4, 5]. Sepsis and septic shock were redefined in 2016 (Sepsis-3), defining sepsis as “life-threatening organ dysfunction caused by a dysregulated host response to infection” [6]. An increase in the sequential organ failure assessment score (SOFA) of ≥ 2 was associated with an in-hospital mortality greater than 10% and proposed for clinical operationalization [6].

As a bedside screening tool for sepsis-related mortality for non-intensive care settings, the quickSOFA (qSOFA) score was introduced to rapidly identify patients with high likelihood of poor sepsis-related outcome, in particular with high risk of sepsis-related mortality [3, 6, 7]. Although, the effort of establishing a new definition and clinical criteria by analyzing large datasets of patient records was widely acknowledged, the validity of these criteria for clinical practice has been questioned [8–12] and their applicability in different settings and subgroups remains unclear [13, 14]. This might particularly apply to specific subgroups such as burn patients [15], surgical patients [16], and hematological cancer patients [14, 17].

Cancer patients and particularly patients with hematological malignancies commonly suffer from immunodeficiency resulting from the underlying disease and/or from immunosuppressive therapy and are therefore at high risk of infection resulting in sepsis [18, 19]. Importantly, population and register-based studies indicate that up to one in five patients admitted to intensive care units (ICU) suffer from underlying malignancies and that sepsis is the leading cause for ICU admission in patients with hematologic malignancies [18, 20–22], although the validity of sepsis criteria in hematological cancer patients remains unclear.

We therefore analyzed the discriminative accuracy of the Sepsis-3 scores and the systemic inflammatory response syndrome (SIRS) criteria for sepsis and in-hospital mortality in a multicenter cohort of hematological cancer patients. The primary aim of this study was to assess the impact of different sepsis scores in discriminating sepsis and in-hospital mortality and to validate their use in these patients admitted to ICU or a regular hematology ward.

## Methods

This was a retrospective, multicenter study of 450 adult patients (≥ 18 years) with hematological cancer admitted between 2013 and 2018 to a regular ward specialized in hematology or the ICU at one of the following three large academic tertiary care hospitals in Germany (University Hospital of Cologne, University Hospital Magdeburg, University Hospital Essen). The patients were admitted for application of therapy or for treatment of complications consecutive to their underlying disease or therapy such as fever and suspected infection. For the ICU patients included in the study, patients with hematological cancer consecutively admitted to an ICU at one of the three centers were included in the study irrespective of the suspected diagnosis at transfer to the ICU. Patients on the normal hematology ward of one of the three centers were included, when an infection was suspected, suspected infection being defined according to current Surviving Sepsis Campaign guidelines and recommendations (administration of antibiotics excluding prophylactic treatment and sampling of body fluid for culturing) [2, 6]. For patients with multiple hospitalizations, we only included their first admission.

The following clinical data were taken from patients’ medical records documented at admission to ICU or when an infection was suspected: patient age, gender, underlying disease and current status of the malignancy (newly diagnosed, partial or complete remission, stable disease, progressive disease), data on hematopoietic stem cell transplant (HSCT) and source of infection, physiological and laboratory data to determine the sepsis scores such as the SIRS criteria [23], the SOFA and the qSOFA score [6], severity indicators such as the Acute Physiology, Age, Chronic Health Evaluation II (APACHE II) score [24], Glasgow Coma Scale (GCS) [25], acute kidney failure and graft-versus-host disease, measures of outcome such as in-hospital mortality, ICU mortality, hospital length of stay and transfer to ICU. Additionally, lactate levels, administration of vasopressors, and mean arterial pressure (MAP) were collected to determine septic shock according to the Sepsis-3 definition from 2016 [3].

According to current Surviving Sepsis Campaign guidelines and recommendations [2, 6], all patients who received antibiotics (excluding prophylactic treatment) and underwent sampling of body fluids for culturing were defined as patients with suspected infection [6], and patients with “life-threatening organ dysfunction caused by a dysregulated host response to infection” were defined as patients with sepsis [6]. For each scoring system, the standard threshold of ≥ 2 points was applied for a positive SIRS, SOFA, and qSOFA score [6, 26]. The baseline SOFA score to differentiate between preexisting and acute changes in organ dysfunction in order to calculate the acute increase in SOFA was assessed with the help of prior laboratory values, the impact of the underlying disease and its therapies. In case of missing data to determine the SIRS, SOFA, and qSOFA score, the patients were excluded from further analysis of diagnostic accuracy. Patients with sepsis and a vasopressor requirement to maintain a MAP of ≥ 65 mmHg and a serum lactate level > 2 mmol/L in the absence of hypovolemia were diagnosed with septic shock according to the Sepsis-3 definition [6].

### Statistical analysis

Dichotomous variables were presented as absolute numbers and percentages and continuous variables as medians and interquartile ranges (IQR). The Mann-Whitney *U* test was used to compare continuous variables between the two groups “patients with sepsis” and “patients without sepsis” after testing for normal distribution using the Shapiro-Wilk test. Categorical binary variables were compared by the chi-squared test. Two-sided *p* values < 0.05 were required for statistical significance.

By applying the definition of sepsis as “life-threatening organ dysfunction caused by a dysregulated host response to infection” [6] as reference, the scores were evaluated. In order to assess the accuracy of the three investigated sepsis criteria, SIRS, SOFA, and qSOFA, regarding sepsis and in-hospital mortality, sensitivity, specificity, predictive values, likelihood ratios (LR), and the Youden’s Index were calculated according to standard formulas as previously described, and the area under receiver operating characteristic (AUROC) curves were determined [27–30]. Pairwise comparison of receiver operating characteristic (ROC) curves was conducted in order to test statistical significance. To further assess the association between sepsis scoring systems and mortality, we also determined the in-hospital mortality for patients with positive and negative sepsis scores and calculated the risk ratio (RR) for each sepsis criterion. All statistical analyses were two-tailed and calculated with Stata/SE 13.

## Results

### Study population

A total of 450 patients with hematological malignancies were included from ICUs or regular hematology wards of three German tertiary academic hospitals. The median age was 58 years (IQR, 46–66 years), 61% were male, 44% had leukemia, 31% had lymphoma, 18% suffered from myeloma, and 4% from myelodysplastic syndrome. Nearly half the population was in partial or complete remission, about one in four patients were newly diagnosed and about one in five patients suffered from disease progression at admission. About 40% had received a HSCT, of which half the patients an allogeneic (49%; Table 1).

**Table 1:** Demographics and clinical characteristics of the cohort

Variable	Total cohort (*n* = 450)	Patients with sepsis (*n* = 180)	Patients without sepsis (*n* = 270)	*p* value
Demographics				
Age in years, median (IQR)	58 (46–66)	58 (44.5–66)	59 (46–66)	0.876
Male gender	274 (60.9)	106 (58.9)	168 (62.2)	0.478
Underlying malignancy				0.177
Non-Hodgkin’s lymphoma	113 (25.1)	39 (21.7)	74 (27.4)	
Hodgkin lymphoma	25 (5.6)	11 (6.1)	14 (5.2)	
Acute myeloid leukemia	127 (28.2)	52 (28.9)	75 (27.8)	
Acute lymphocytic leukemia	36 (8.0)	15 (8.3)	21 (7.8)	
Multiple myeloma	80 (17.8)	30 (16.7)	50 (18.5)	
Chronic myeloid leukemia	9 (2.0)	6 (3.3)	3 (1.1)	
Chronic lymphocytic leukemia	25 (5.6)	8 (4.4)	17 (6.3)	
Myelodysplastic syndrome	18 (4.0)	10 (5.6)	8 (3.0)	
Other	17 (3.8)	9 (5.0)	8 (3.0)	
Disease status at admission				0.184
Newly diagnosed	118 (26.2)	40 (22.2)	78 (28.9)	
Partial or complete remission	202 (44.9)	86 (47.8)	116 (43.0)	
Stable disease	14 (3.1)	5 (2.8)	9 (3.3)	
Progressive disease	91 (20.2)	34 (18.9)	57 (21.1)	
Unknown	25 (5.6)	15 (8.3)	10 (3.7)	
Hematopoietic stem cell transplant	184 (40.9)	82 (45.6)	102 (37.8)	0.100
Allogeneic	87 (47.3)	45 (54.9)	42 (41.2)	
Autologous	94 (51.1)	36 (43.9)	58 (56.9)	
Infection site (patients with sepsis)				
Respiratory tract		58 (32.2)		
Central line-related		17 (9.4)		
Gastrointestinal tract		16 (8.9)		
≥ 3 sepsis foci		5 (2.8)		
Other		18 (10.0)		
Unknown		66 (36.7)		
Laboratory and physiological data^a^, median (IQR)				
White blood cell count (× 10^9^/L)	0.90 (0.09–6.23)	0.60 (0.06–6.39)	1.08 (0.14–5.8)	0.093
Platelet count (× 10^9^/L)	31.0 (15–78)	26.5 (13–65)	35.0 (16–91)	*0.002*
Creatinine (μmol/L)	89.3 (62.8–136.1)	103.4 (67.2–217.5)	84.0 (61.9–116.7)	*< 0.001*
Bilirubin (μmol/L)	13.7 (8.6–24.0)	17.1 (8.6–46.2)	12.0 (6.8–18.8)	*< 0.001*
MAP (mmHg)	80 (70–90)	73 (65–87)	83 (74–91)	*< 0.001*
Temperature (°C)	38.1 (37.2–38.5)	38.2 (37.0–38.7)	38.1 (37.6–38.4)	0.482
Systolic pressure (mmHg)	120 (100–130)	110 (90–125)	120 (106–132)	*< 0.001*
Heart rate (bpm)	100 (84–116)	108 (88–128)	96 (84–106)	*< 0.001*
Lactate (mmol/L), ICU only^b^	1.9 (1.16–3.1)	2.0 (1.21–3.7)	1.84 (1.1–2.7)	0.226

Data are presented as absolute number (%), unless otherwise indicated

*p* value between the respective variable and sepsis with a significance level of *p* value < 0.05, significant differences are shown in italics

*IQR* interquartile range, *MAP* mean arterial pressure

^a^At least 98% of the cohort contributed to the presented data

^b^At least 90% of the cohort contributed to the presented data

One hundred eighty patients (40%) presented with sepsis of which 101 (56%) were treated on ICU and an additional 27 patients (15%) were later transferred to an ICU (Table 2). The two subgroups of patients (sepsis vs. no sepsis) had a similar distribution regarding age and gender but differed significantly in platelet count, creatinine and bilirubin. Patients with sepsis were more likely to present with acute kidney failure, lower MAP, and higher heartrates at admission. The measured body temperature was not significantly different between the two groups. The most common source of infection in patients with sepsis was the respiratory tract (32%) followed by central line-associated infections and the gastrointestinal tract both in 9% of the patients (Tables 1 and 2).

**Table 2:** Outcomes and severity indicators

Variable	Total cohort (*n* = 450)	Patients with sepsis (*n* = 180)	Patients without sepsis (*n* = 270)	*p* value
Severity Indicators				
Microbes detected^a^	202 (45.5)	112 (62.9)	90 (33.8)	*< 0.001*
Acute kidney failure^c^	82 (22.2)	46 (31.5)	36 (16.1)	*< 0.001*
Tumor lysis syndrome^c^	6 (1.6)	3 (2.1)	3 (1.4)	0.590
Graft-versus-host disease^c^	20 (5.4)	7 (4.9)	13 (5.8)	0.708
Glasgow Coma Scale^d^, mean (SD)	14.0 (2.9)	13.3 (3.6)	14.4 (2.2)	*< 0.001*
Outcomes				
Overall in-hospital mortality	109 (24.3)	71 (39.7)	38 (14.1)	*< 0.001*
ICU mortality	80 (44.4)	52 (51.5)	28 (35.0)	*0.023*
Length of stay^e^, median days (IQR)	26 (16–41)	28 (15–44)	25.5 (17–40)	0.554
Transfer from regular ward to ICU^e^	49 (18.3)	27 (34.2)	22 (11.6)	*0.005*
Sepsis scores^f^				
SIRS ≥ 2	351 (80.1), *n* = 438	150 (85.7), *n* = 175	201 (76.4), *n* = 263	*0.017*
SOFA ≥ 2	173 (42.2), *n* = 410	112 (64.0), *n* = 175	61 (26.0), *n* = 235	*< 0.001*
qSOFA ≥ 2	90 (22.0), *n* = 410	68 (41.5), *n* = 164	22 (8.9), *n* = 246	*< 0.001*
Septic shock	20 (4.5), *n* = 441	20 (11.7), *n* = 171	–	*< 0.001*
SIRS < 2	3 (15.0)	3 (15.0)		
SOFA < 2	2 (10.0)	2 (10.0)		
qSOFA < 2	4 (20.0)	4 (20.0)		
ICU (*n* = 181)				
Severity and septic shock indicators				
APACHE II^d^, mean (SD)	22.3 (8.0)	24.1 (8.0)	20 (7.5)	*< 0.001*
Hypotension (MAP < 65 mmHg)^a^	46 (25.8)	33 (33.3)	13 (16.5)	*< 0.001*
Use of vasopressors^a^	97 (54.2)	65 (65.6)	32 (40.0)	*< 0.001*
Lactate > 2 mmol/L^b^	78 (47.0)	46 (48.9)	32 (44.4)	0.226

Data are presented as absolute number (%), unless otherwise indicated

*p* value between the respective variable and sepsis with a significance level of *p* value < 0.05, significant differences are shown in italics

*APACHE II* Acute Physiology, Age, Chronic Health Evaluation II, *ICU* intensive care unit, *IQR* interquartile range, *MAP* mean arterial pressure, *SD* standard deviation, *SIRS* systemic inflammatory response syndrome, *SOFA* sequential (sepsis-related) organ failure assessment, *qSOFA* quick SOFA

^a^At least 98% of the cohort contributed to the presented data

^b^At least 90% of the cohort contributed to the presented data

^c^At least 80% of the cohort contributed to the presented data

^d^At least 95% of the cohort contributed to the presented data

^e^At least 99% of the cohort contributed to the presented data

^f^For SIRS 97%, for SOFA and qSOFA 91%, and for septic shock 98% of the cohort contributed to the presented data

### Performance of SIRS, SOFA and qSOFA criteria

Within the group of patients with sepsis, 86% (95% confidence interval [95% CI], 80–90%) of the patients presented with a ≥ 2 positive SIRS criteria, 64% (95% CI, 57–71%) of the patients with a SOFA score ≥ 2, 42% (95% CI, 34–49%) of the patients with a qSOFA score ≥ 2, and 20 patients (12%) fulfilled the Sepsis-3 criteria of septic shock. Thus, the false-negative rate for the SIRS criteria was 14%, for the SOFA score 36%, and for the qSOFA score 59%. Within the group of patients not suffering from sepsis, 76% of the patients presented with ≥ 2 SIRS criteria, 26% with a SOFA score ≥ 2, and 9% with a qSOFA score ≥ 2, representing the number of false positives for each score, respectively (Table 2, 2×2 contingency tables and flow charts for each score are provided in Additional files 1 and 2).

**Fig. 1:**
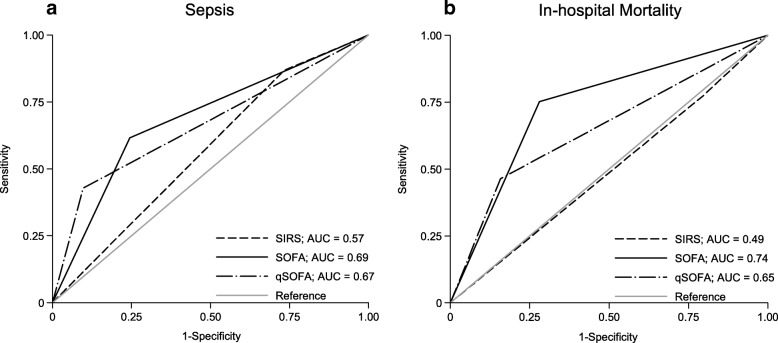
Receiver operating characteristic (ROC) curves (unadjusted) for **a** sepsis and **b** in-hospital mortality. The areas under the ROC curves (AUROCs) for panel **a** are SIRS, 0.57 (95% confidence interval [CI], 0.53–0.61), *p* < 0.001; SOFA, 0.69 (95% CI, 0.64–0.73), *p* < 0.001; qSOFA, 0.67 (95% CI, 0.62–0.71), *p* < 0.001, and for panel **b** SIRS, 0.49 (95% CI, 0.44–0.54), *p* < 0.001; SOFA, 0.74 (95% CI, 0.69–0.79), *p* < 0.001; qSOFA, 0.65 (95% CI, 0.60–0.71), *p* < 0.001. *AUC* area under the curve, *SIRS* systemic inflammatory response syndrome, *SOFA* sequential (sepsis-related) organ failure assessment, *qSOFA* quick SOFA

Prognostic performances of the three investigated sepsis criteria for the total cohort are reported in Table 3. The Youden’s Index as an indicator for the performance of dichotomous diagnostic tests was 0.093 for SIRS, 0.380 for SOFA, and 0.325 for qSOFA. The AUROC values for discrimination of sepsis were 0.57 (95% confidence interval [95% CI], 0.53–0.61; *p* < 0.001) for SIRS, 0.69 (95% CI, 0.64–0.73; *p* < 0.001) for SOFA, and 0.67 (95% CI, 0.62–0.71; *p* < 0.001) for qSOFA (Fig. 1). When comparing the ROC curves of SOFA with qSOFA, the areas are not significantly different (*p* value = 0.309); however, comparing the ROC curves of SOFA or qSOFA with SIRS, respectively, the areas are significantly different (both *p* values < 0.001).

**Table 3:** Performance of SIRS, SOFA and qSOFA scores

Diagnostic accuracy and outcome measures	SIRS	SOFA	qSOFA
Diagnosis of sepsis			
Total cohort (*n* = 450)^f^			
Sensitivity, % (95% CI)	85.7 (79.8–90.1)	64.0 (56.7–70.7)	41.5 (34.2–49.1)
Specificity, % (95% CI)	23.6 (18.9–29.1)	74.0 (68.1–79.2)	91.1 (86.8–94.0)
Youden’s Index	0.093	0.380	0.325
PPV, % (95% CI)	42.7 (37.5–48.1)	64.7 (57.1–71.7)	75.6 (65.2–83.7)
NPV, % (95% CI)	71.3 (60.4–80.2)	73.4 (67.2–78.8)	70.0 (64.6–74.9)
AUROC (95% CI)	0.57 (0.53–0.61); *p* < 0.001	0.69 (0.64–0.73); *p* < 0.001	0.67 (0.62–0.71); *p* < 0.001
In-hospital mortality			
Total cohort (*n* = 450)^*f*^			
Sensitivity, % (95% CI)	79.2 (70.6–85.9)	76.2 (67.2–83.3)	45.0 (35.6–54.8)
Specificity, % (95% CI)	19.6 (15.7–24.2)	69.5 (64.1–74.4)	85.5 (81.1–89.0)
Youden’s Index	–	0.456	0.304
AUROC (95% CI)	0.49 (0.44–0.54); *p* < 0.001	0.74 (0.69–0.79); *p* < 0.001	0.65 (0.60–0.71); *p* < 0.001
Mortality if score ≥ 2	84 (23.9), *n* = 351	80 (46.2), *n* = 173	45 (50.0), *n* = 90
Mortality if score < 2	22 (25.3), *n* = 87	25 (10.5), *n* = 237	55 (17.2), *n* = 320
Risk ratio (95% CI)	0.95 (0.63–1.42); *p* = 0.792	4.38 (2.93–6.57); *p* < 0.001	2.91 (2.12–3.99); *p* < 0.001
Patients with sepsis (*n* = 180)			
Mortality if score ≥ 2	58 (38.7), *n* = 150	63 (56.3), *n* = 112	33 (48.5), *n* = 68
Mortality if score < 2	10 (40.0), *n* = 25	7 (11.1), *n* = 63	32 (33.3), *n* = 96
Risk ratio (95% CI)	0.97 (0.57–1.63); *p* = 0.899	5.06 (2.47–10.37); *p* < 0.001	1.44 (0.99–2.09); *p* = 0.056
Patients without sepsis (*n* = 270)			
Mortality if score ≥ 2	26 (12.9), *n* = 201	17 (27.9), *n* = 61	12 (54.5), *n* = 22
Mortality if score < 2	12 (19.4), *n* = 62	18 (10.3), *n* = 174	23 (10.3), *n* = 224
Risk ratio (95% CI)	0.67 (0.36–1.24) *p* = 0.209	2.69 (1.49–4.89); *p* = 0.001	5.31 (3.08–9.15); *p* < 0.001
ICU patients with sepsis (*n* = 101)			
Mortality if score ≥ 2	41 (50.0), *n* = 82	48 (56.5), *n* = 85	28 (57.1), *n* = 49
Mortality if score < 2	10 (58.8), n = 17	3 (25.0), *n* = 12	23 (48.9), *n* = 47
Risk ratio (95% CI)	0.85 (0.54–1.34); *p* = 0.508	2.26 (0.83–6.13); *p* = 0.041	1.17 (0.80–1.71); *p* = 0.421

^f^For SIRS 97%, for SOFA and qSOFA 91%, and for septic shock 98% of the hematological cohort contributed to the presented data

*AUROC* area under receiver operating characteristic, *CI* confidence interval, *ICU* intensive care unit, *NPV* negative predictive value, *PPV* positive predictive value, *SIRS* systemic inflammatory response syndrome, *SOFA* sequential (sepsis-related) organ failure assessment, *qSOFA* quick SOFA

### In-hospital mortality

For the prediction of in-hospital mortality, SIRS had a sensitivity of 79% (95% CI, 71–86%) and a specificity of 20% (95% CI, 16–24%), SOFA had a sensitivity of 76% (95% CI, 67–83%) and a specificity of 70% (95% CI, 64–74%), and for qSOFA, the sensitivity was 45% (95% CI, 36–55%) and the specificity was 86% (95% CI, 81–89%). The positive likelihood ratio was 3.1 (95% CI, 2.2–4.4) for qSOFA and 2.5 (95% CI, 2.1–3.1) for SOFA. The negative predictive value was 83% for qSOFA and 90% for SOFA, respectively. Figure 1 and Table 3 show the AUROCs and values for the prediction of in-hospital mortality by the three sepsis scores. The highest AUROCs were determined for SOFA (0.74 [95% CI, 0.69–0.79]; *p* < 0.001) and qSOFA score (0.65 [95% CI, 0.60–0.71]; *p* < 0.001) compared to SIRS (0.49 [95% CI, 0.44–0.54]; *p* < 0.001). When comparing the ROC curves of SOFA with qSOFA regarding prediction of in-hospital mortality, there was a significant difference (*p* value = 0.005).

In the whole cohort, including ICU and non-ICU patients, in-hospital mortality was 24% with an ICU mortality of 44%. Of all patients with sepsis, 40% died compared to 14% of all patients without sepsis (*p* < 0.001; Table 2).

Focusing on patients with sepsis, the mortality rate in patients with positive and negative SIRS scores was 39% vs. 40% (*p* = 0.899), respectively. For patients with a qSOFA score ≥ 2, mortality was 49% compared to 33% for those with a qSOFA score < 2 (*p* = 0.056), and for the SOFA score 56% vs. 11% (*p* < 0.001), respectively. Thus, the mortality rate for sepsis patients with falsely negative scores was lowest (11%) for the SOFA score. Patients with sepsis with SOFA scores ≥ 2 were about five times more likely to die (risk ratio 5.06 [95% CI, 2.47–10.37]) compared to patients with SOFA scores < 2. Regarding qSOFA, patients with a qSOFA score ≥ 2 were about 1.5 times more likely to die compared to patients with a score < 2 (risk ratio 1.44 [95% CI, 0.99–2.09]). In contrast, there was little difference in mortality in patients with ≥ 2 SIRS scores compared to patients with SIRS scores < 2 (risk ratio 0.97 [95% CI, 0.57–1.63]). In the group of patients with sepsis treated on the ICU, patients with ≥ 2 SOFA scores were more than twice as likely to die as patients with SOFA scores < 2 (risk ratio 2.26 [95% CI, 0.83–6.13]), being the only risk ratio with a significance level < 0.05 in this group (Table 3).

## Discussion

As population-based studies show, about one in five patients in the ICU has cancer and the proportion of ICU patients suffering from cancer is expected to increase over the next decades [18, 21, 31]. Patients with hematological malignancies are at particular risk of requiring ICU treatment as therapy- or disease-related immunodeficiency is common in hematological cancer patients frequently leading to infection and sepsis [20, 31]. At the same time, patients with neutropenia and immunodeficiency frequently do not exhibit fever, pus, and other symptoms associated with the infection; thus, especially in patients with hematological malignancies and sepsis, it can be difficult to determine the site of infection (37%). In addition, many patients suffer from polymicrobial infection originating from mucosal barrier injury, which is difficult to diagnose with conventional methods and criteria.

Although the accurate definition and early diagnosis of sepsis indisputably improve patient survival, there is a considerable debate about new definitions and criteria for sepsis [2–13, 15–17]. Moreover, the discriminatory capacity of different criteria and scores, such as SIRS, SOFA, and qSOFA, for identifying sepsis and predicting in-hospital mortality in hematological cancer patients remains unclear.

The focus of our study was the evaluation of the diagnostic validity of these criteria for the diagnosis of sepsis and the prediction of high risk of in-hospital mortality in a multicentric population of hematological cancer patients. We studied the criteria in analogy to the Sepsis-III study focusing on the criteria and their (bedside) validity, rather than on changes of inflammation markers, which has been studied previously by several groups [32, 33].

In our study, we found a higher non-ICU mortality of 11% compared to the mortality of 3% reported in the original Sepsis-3 study from Seymour and colleagues in a general population [7]. ICU mortality of hematological cancer patients in our study was 44% and even increased up to 50% if patients suffered from sepsis, which is within the reported range for hematological cancer ICU patients of 34–68% [34] and slightly higher than reported for the largest published hematological cancer cohort by Azoulay et al. (39%) [20]. Costa et al. studied a single-center cohort of 450 cancer patients admitted to the ICU, including about 19% of patients with hematological malignancies, and reported an overall mortality of 39% [17]. Soares and colleagues studied a very large multicenter retrospective cohort of 9946 cancer patients, including about 10% (*n* = 990) of patients with hematologic malignancies, reporting ICU and in-hospital mortality rates of 16% and 25%, respectively [21]. These findings are well in line with previous reports, indicating a higher mortality of hematological cancer ICU patients compared to studies including patients with solid tumors, which often include a higher proportion of patients with post-surgical surveillance [20, 21, 31]. Neutropenia, which is common in hematological cancer patients, but less in patients with solid tumors, might be an important factor. Although earlier data were conflicting, neutropenia was independently associated with an increased mortality in a recent meta-analysis including 4149 (55%) patients with hematological cancer [35], which might contribute to the high mortality we observed.

Regarding the validity and performance of different sepsis scores in our cohort, we found that more than one in six patients (17%) with sepsis did not meet two or more SIRS criteria. This proportion of “SIRS-negative” sepsis patients in our study was slightly higher than previously reported by Kaukonen et al. (12%) in a population-based ICU cohort [36]. Costa et al. reported exclusively on cancer patients with a suspected infection admitted to the ICU and observed about one fifth of patients with sepsis who met less than two SIRS criteria (23%) [17]. However, both studies did not include information on the respective criteria in patients without sepsis [17, 36]. Interestingly, the majority of patients in our study without sepsis met two or more SIRS criteria (76%). In those false-positive patients, almost all patients (90%) had abnormal white blood cell counts (< 4 × 10^9^/L or > 12 × 10^9^/L), which were responsible for most positive SIRS scores in patients without sepsis. This observation of a high proportion of “SIRS-positive” patients without sepsis was similarly reported by several authors in different populations including ICU and non-ICU patients [13]. Therefore SIRS criteria are of limited use in diagnosing and screening for sepsis in hematological cancer patients as abnormal white blood cell counts are a common disease- or therapy-related observation in these populations.

Both scores, SOFA and qSOFA, had a lower proportion of false-positive patients; however, the proportion of patients with sepsis, who did not meet two or more of the criteria, also increased. Thirty-six percent of the patients with sepsis did not meet a positive SOFA score (two or more) and more than half of the patients with sepsis (59%) had less than two of three qSOFA criteria (Table 3). The qSOFA was mainly negative due to a GCS of 15 (in 92% of the patients) and a systolic blood pressure above 100 mmHg (73% of the patients).

Yet, the mortality rate for the 36% of sepsis patients with false-negative SOFA scores was low (11%) compared to the respective mortality rates for SIRS (40%) and qSOFA (33%).

Overall, we found that SOFA was more accurate than qSOFA or SIRS in discriminating hematological cancer patients with sepsis from patients without sepsis (SOFA, AUROC 0.69 [95% CI, 0.64–0.73], *p* < 0.001; qSOFA, AUROC 0.67 [95% CI, 0.62–0.71], *p* < 0.001; SIRS, AUROC 0.57 [95% CI, 0.53–0.61], *p* < 0.001; Table 3). Focusing on ICU patients, the sensitivity in hematological cancer patients was highest for SOFA (88%) and the false-negative rate for ICU patients with sepsis was lower (12%) compared to the other two scoring systems.

Moreover, in-hospital mortality in hematological cancer patients with sepsis was about fivefold higher in “SOFA-positive” patients compared to patients with a SOFA score below two (risk ratio 5.06 [95% CI, 2.47–10.37]; *p* < 0.001). The discrimination of in-hospital mortality using SOFA (AUROC 0.74 [95% CI, 0.69–0.79]; *p* < 0.001) was significantly higher compared with SIRS criteria (AUROC 0.49 [95% CI, 0.44–0.54]; *p* < 0.001), for which the mortality rate was 40% in “SIRS-negative” patients and about 39% in “SIRS-positive” patients (Table 3). Similarly, several authors previously suggested SOFA as predictor of ICU mortality in general ICU populations with suspected infection, and the relationship between SOFA and mortality has been investigated in a variety of subgroups [7, 17, 37–40].

Importantly, there are significant limitations to the clinical use of the SOFA score, as it requires laboratory measurements and is time-consuming to calculate. However, our results in a hematological cancer population including non-ICU patients suggest that SIRS criteria are not accurate in hematological cancer patients and that also qSOFA, which has been proposed as an effective way of raising suspicion of sepsis on the regular hematology ward might not be applicable in hematological cancer patients, as the number of qSOFA-negative sepsis patients with high mortality was considerable in our study.

Strengths of our study are the large number of hematological cancer patients and the multicentric design including patients from three tertiary academic hospitals. Furthermore, our cohort included a broad range of hematological cancer patients regarding disease status, HSCT, and organ dysfunction. The main limitation of the study was the retrospective study design with data acquisition possibly being prone to bias. In addition, some of the data needed to calculate the SOFA score could not be collected for non-ICU patients. Furthermore, despite the fact that we found a strong association of several characteristics with mortality including indicators of organ failure, we could not determine the impact of each single factor in a multivariate analysis, as the heterogeneity of our study population was too high regarding the sample size. A multivariate analysis would require a much higher number of patients to provide reproducible results and was not a focus of our study.

## Conclusions

In conclusion, we found that in our cohort of patients with hematological malignancies, an increase in SOFA score of ≥ 2 had superior prognostic accuracy for discriminating sepsis and estimating in-hospital mortality compared with qSOFA or SIRS criteria. Especially on intensive care, we observed limited validity of SIRS and qSOFA in identifying hematological patients with sepsis and at high risk of death. The significant proportion of sepsis patients with a negative score, irrespective of the scoring system used, underlines the need for a clinical diagnosis of sepsis particularly in hematological cancer patients.

## Additional files

Additional file 1: 2x2 contingency tables for a SIRS criteria, b SOFA score and c qSOFA score. (DOCX 23 kb)

Additional file 2: Flow charts for a SIRS criteria, b SOFA score and c qSOFA score. (PPTX 44 kb)

## Data Availability

The datasets used and/or analyzed during the current study are available from the corresponding author on reasonable request.

## Abbreviations

APACHE II: Acute Physiology, Age, Chronic Health Evaluation II
AUROC: Area under receiver operating characteristic
CI: Confidence interval
GCS: Glasgow coma scale
HSCT: Hematopoietic stem cell transplant
ICU: Intensive care unit
IQR: Interquartile range
LOS: Length of stay
LR: Likelihood ratio
MAP: Mean arterial pressure
qSOFA: Quick sequential (sepsis-related) organ failure assessment
ROC: Receiver operating characteristic
RR: Risk ratio
SD: Standard deviation
SIRS: Systemic inflammatory response syndrome
SOFA: Sequential (sepsis-related) organ failure assessment
