# Supplementary material for: Prognostic accuracy of SOFA, qSOFA and SIRS criteria in hematological cancer patients: a retrospective multicenter study
Source: J Intensive Care. 2019 Aug 7;7:41. doi: 10.1186/s40560-019-0396-y (PMC6686367; doi:10.1186/s40560-019-0396-y)
Supplement: Supplementary file 1 — 2x2 contingency tables for a SIRS criteria, b SOFA score and c qSOFA score. (DOCX 23 kb) [file 40560_2019_396_MOESM1_ESM.docx]

**Supplementary Material 1:** 2x2 contingency tables for **a** SIRS criteria **b** SOFA score and **c** qSOFA score

**a**

|  | Hematological patients | | |
| --- | --- | --- | --- |
| **SIRS Score** | with sepsis | without sepsis | Total |
| Positive  (≥2 criteria) | 150 | 201 | 351 |
| Negative  (<2 criteria) | 25 | 62 | 87 |
| Total | 175 | 263 | 438 |

**b**

|  | Hematological patients | | |
| --- | --- | --- | --- |
| **SOFA Score** | with sepsis | without sepsis | Total |
| Positive  (≥2 criteria) | 112 | 61 | 173 |
| Negative  (<2 criteria) | 63 | 174 | 237 |
| Total | 175 | 235 | 410 |

**c**

|  | Hematological patients | | |
| --- | --- | --- | --- |
| **qSOFA Score** | with sepsis | without sepsis | Total |
| Positive  (≥2 criteria) | 68 | 22 | 90 |
| Negative  (<2 criteria) | 96 | 224 | 320 |
| Total | 164 | 246 | 410 |
