# Supplementary material for: Prognostic accuracy of SOFA, qSOFA and SIRS criteria in hematological cancer patients: a retrospective multicenter study
Source: J Intensive Care. 2019 Aug 7;7:41. doi: 10.1186/s40560-019-0396-y (PMC6686367; doi:10.1186/s40560-019-0396-y)
Supplement: Supplementary file 2 — Flow charts for a SIRS criteria, b SOFA score and c qSOFA score. (PPTX 44 kb) [file 40560_2019_396_MOESM2_ESM.pptx]

## Slide 1
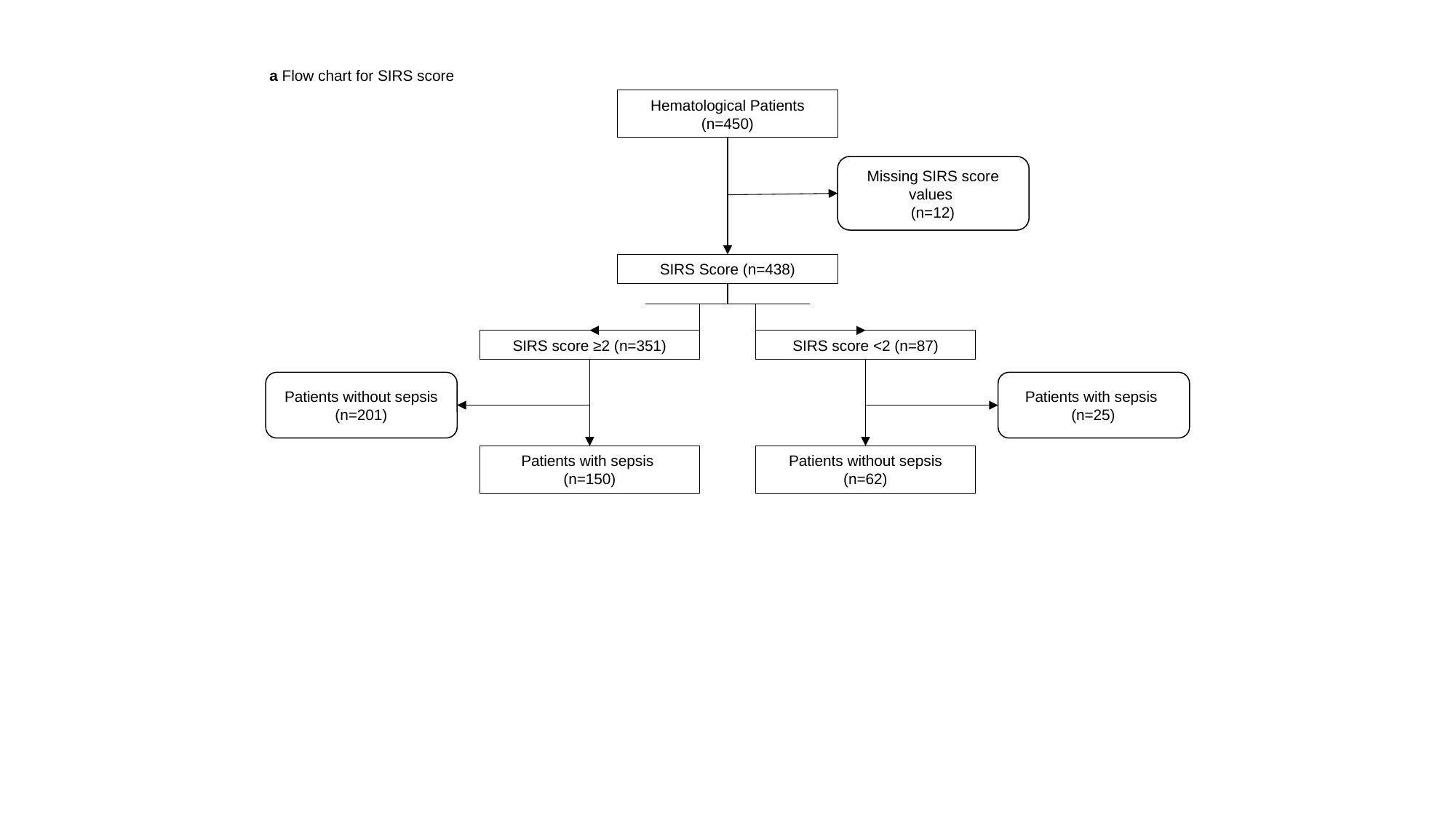

a Flow chart for SIRS score
Hematological Patients (n=450)
Missing SIRS score values
(n=12)
SIRS Score (n=438)
SIRS score ≥2 (n=351)
SIRS score <2 (n=87)
Patients without sepsis
(n=201)
Patients with sepsis
(n=25)
Patients without sepsis (n=62)
Patients with sepsis
(n=150)

## Slide 2
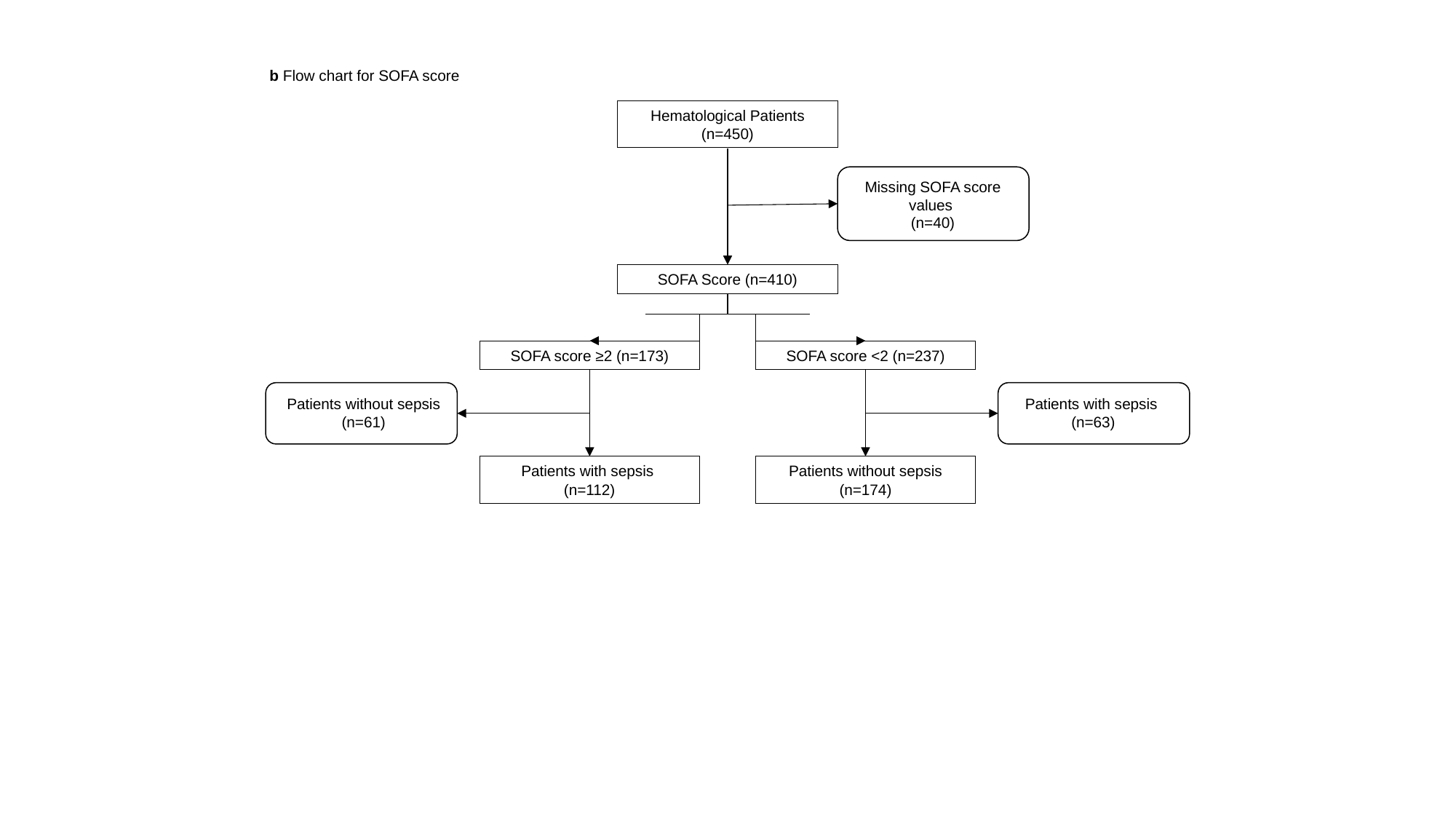

b Flow chart for SOFA score
Hematological Patients (n=450)
Missing SOFA score values
(n=40)
SOFA Score (n=410)
SOFA score ≥2 (n=173)
SOFA score <2 (n=237)
Patients without sepsis
(n=61)
Patients with sepsis
(n=63)
Patients without sepsis (n=174)
Patients with sepsis
(n=112)

## Slide 3
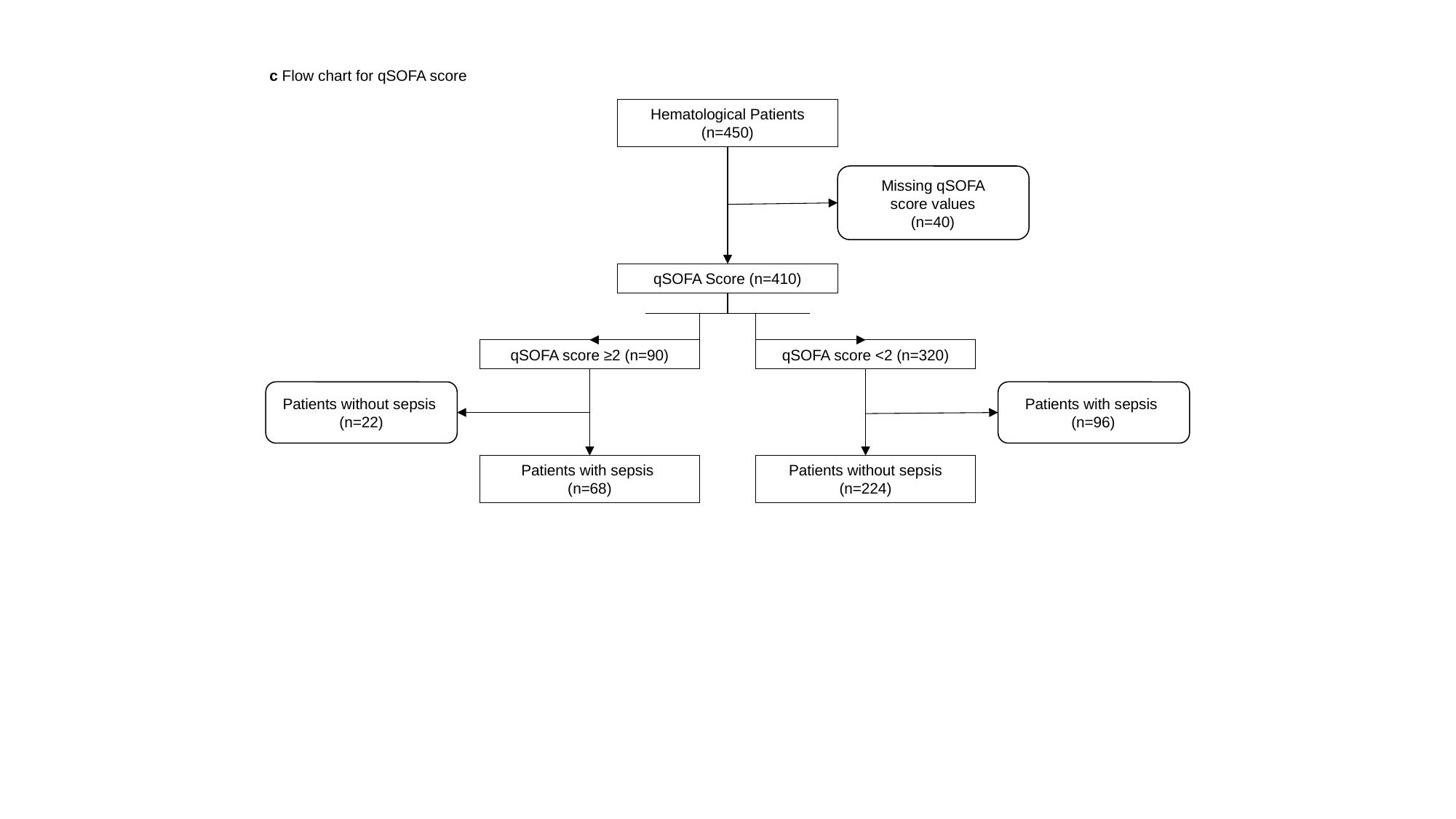

c Flow chart for qSOFA score
Hematological Patients (n=450)
Missing qSOFA score values
(n=40)
qSOFA Score (n=410)
qSOFA score ≥2 (n=90)
qSOFA score <2 (n=320)
Patients without sepsis
(n=22)
Patients with sepsis
(n=96)
Patients without sepsis (n=224)
Patients with sepsis
(n=68)
